# Contribution of subcutaneous abdominal fat on ultrasonography to carotid atherosclerosis in patients with type 2 diabetes mellitus

**DOI:** 10.1186/1475-2840-13-67

**Published:** 2014-03-28

**Authors:** Chan-Hee Jung, Bo-Yeon Kim, Kyu-Jin Kim, Sang-Hee Jung, Chul-Hee Kim, Sung-Koo Kang, Ji-Oh Mok

**Affiliations:** 1 Division of Endocrinology and Metabolism, Department of Internal Medicine, Soonchunhyang University College of Medicine, #170 Jomaru-ro, Wonmi-gu, Bucheon-si, Gyeonggi-do 420-767, South Korea; 2 Department of Obstetrics and Gynecology, Cha University School of Medicine, Bundang, South Korea

**Keywords:** Carotid atherosclerosis, Subcutaneous abdominal fat, Subcutaneous fat thickness, Visceral fat thickness, Ultrasonography, Type 2 diabetes mellitus

## Abstract

**Background:**

Whereas visceral abdominal adipose tissue (VAT) is associated with cardiometabolic risk, there is debate regarding the role of subcutaneous abdominal adipose tissue (SAT). The aim of this study was to investigate the relationships of subcutaneous and visceral abdominal fat with carotid atherosclerosis in patients with type 2 diabetes mellitus (T2DM).

**Methods:**

A total of 234 patients (men 131, women 103, mean age: 53 years) with T2DM were enrolled. Carotid intima-media thickness (CIMT), abdominal subcutaneous fat thickness (SFT) and visceral fat thickness (VFT) were assessed by high-resolution B-mode ultrasonography (US).

**Results:**

Compared to women, men had significantly higher VFT and lower SFT (p = 0.002, p = 0.04, respectively). In partial correlation coefficient analyses between CIMT and abdominal fat thickness after adjustment for body mass index (BMI), SFT showed a negative correlation with CIMT in men (r = -0.27, p = 0.03). VFT was not correlated with CIMT in either men or women. In women, SFT was not correlated with CIMT (r = -0.01, p = 0.93). VFT/SFT ratio was not correlated with CIMT in either men or women. In multivariate regression analyses adjusted for BMI and other CVD risk factors, SFT but not VFT was independently inversely associated with CIMT in men but not in women (p < 0.001).

**Conclusions:**

SFT assessed by US was inversely associated with carotid atherosclerosis in patients with T2DM, particularly men. Further research into the different roles of the two types of abdominal adipose tissue in both men and women is warranted.

## Background

Obesity is associated with several types of cardiometabolic disturbances and metabolic syndrome (MS) [1]. Moreover, T2DM increases the risks of atherosclerosis and cardiovascular disease (CVD) and is known as a coronary heart disease (CHD) risk equivalent [2]. It is evident that the abdominal fat accumulation plays a central role in the pathogenesis of CVD in obese subjects [1].

Excess visceral abdominal adipose tissue (VAT) and subcutaneous abdominal adipose tissue (SAT) are key contributors to abdominal obesity, but differ in their structural composition, metabolic activity, and functional significance [3]. VAT is clearly associated with increased risks of MS and CVD, whereas there has been much debate regarding the role of SAT [4,5]. Prospective studies have shown that the direction and strength of associations between SAT and CVD risk factors and atherosclerosis are nearly identical to those for VAT [6,7]. In contrast, a few studies have suggested a possible beneficial role for SAT [8]. A previous study in a sample of middle-aged men and women found that higher amounts of subcutaneous abdominal fat are associated with lower levels of subclinical atherosclerosis [9]. It remains unclear if SAT may be risk enhancing or, rather, protective for CVD.

The standard methods of assessing abdominal fat accumulation is computed tomography (CT) and magnetic resonance imaging (MRI) [10,11]. However, several studies report that diverse indices using US are a reliable way of quantifying the visceral fat depot, and ultrasonographic VFT was strongly correlated with visceral adipose tissue by CT [12,13]. Also, reliability of the US for assessing SFT is demonstrated in several studies [14-16].

CIMT is an established marker for subclinical atherosclerosis in the general population and in individuals with diabetes [17,18]. Whereas recent observational studies have reported that abdominal fat accumulation on US might be associated with subclinical atherosclerosis in the general population or in the obese, there have been few studies regarding the relationship between abdominal fat assessed by US and subclinical atherosclerosis in T2DM [13,19,20]. Moreover, no previously published studies investigated the effects of SFT on US to detect carotid atherosclerosis in T2DM. Therefore, the aim of this study was to investigate and compare the relationships of SFT and VFT with carotid atherosclerosis and cardiovascular risk factors in patients with T2DM.

## Methods

### Subjects

Patients with T2DM who visited the diabetes clinic at Soonchunhyang University Bucheon Hospital from January 2012 to December 2012 were recruited. Among them, patients who had undergone thorough evaluations for carotid atherosclerosis and abdominal SFT and VFT were included in this study. Individuals with renal insufficiency, cirrhosis with ascites, known hyperthyroidism or hypothyroidism, chronic infection, malignancy, and were not available of fasting serum samples and clinical data from appropriate medical records were excluded. A total of 234 patients (mean age: 53 years) were included in this study. The alcohol consumption of the subjects was classified as ‘yes or no’. The smoking status of the subjects was classified as being a non-smoker or smoker (former or current). This study was approved by the institutional review board of Soonchunhyang University Bucheon Hospital.

### Biomarker measurements

All subjects underwent standard examination and testing, which included measurements of the concentrations of fasting glucose, glycated hemoglobin (HbA1c), total cholesterol (TC), low density lipoprotein cholesterol (LDL-C), high density lipoprotein cholesterol (HDL-C), triglyceride (TG), high-sensitivity C-reactive protein (hs-CRP), uric acid, and homeostasis model assessment-insulin resistance (HOMA-IR). HbA1c was measured by ion-exchange high-performance liquid chromography (Bio-Rad, Hercules, CA, USA).

The baPWV and ABI were measured by an automated device (VP-1000; Colin, Komaki, Japan) . The PWV and ABI, the blood pressure of both extremities, an electrocardiogram and heart sounds were measured simultaneously. The electrocardiogram electrodes were attached to both wrists and a heart sound microphone was placed on the left sterna border. Cuffs were encircled around both arms and ankles, and the cuffs were attached to both a plethymographic sensor determined the volume pulse form, and the blood pressure was measured from the oscillometric pressure sensor. PWV (cm/s) was defined as the distance between two distinct points (cm) divided by the pulse wave transit time (s). The ABI was defined as the ratio of the systolic blood pressure measured at the ankle to the systolic blood pressure measured at the brachial artery. The insulin resistance status was evaluated by the HOMA-IR index. The HOMA-IR was calculated by the formula: [fasting insulin (uIU/mL) × fasting blood glucose (mmol/L)]/22.5 [21]. The HOMA-IR index was available only for 159 patients who were not receiving exogenous insulin.

Diabetic nephropathy was defined using albuminuria, which was measured by radioimmunoassay (Immunotech). Albumin excretion rate (AER) <20 μg/min or urine albumin <30 mg/g creatinine was categorized as normoalbuminuria, AER in the range of 20–200 μg/min or urine albumin 30–300 mg/g creatinine as microalbuminuria, and AER > 200 μg/min or urine albumin ≥ 300 mg/g creatinine as overt proteinuria. Patients were considered to have nephropathy if they show microalbuminuria or overt proteinuria.

### Abdominal fat and CIMT assessment by US

Patients were weighed in light clothing, and height was measured while barefoot. Body mass index (BMI) was calculated as weight in kilograms divided by the square of height in meters. All anthropometric measurements were obtained by a skilled nurse in our diabetes clinic.

All sonographic measurements of abdominal fat thickness and carotid IMT were performed using a high resolution B-mode US (SSA-660A, Toshiba, Tokyo, Japan) by a single experienced investigator. SFT and VFT were measured in the region 1 cm above the umbilicus using a 12-MHz linear-array probe and a 3.5-MHz convex-array probe, respectively. Patients were examined during the expiratory phase of quiet respiration, and the transducer was applied on the body surface without undue pressure. SFT was defined as the maximal thickness of the fat tissue layer between the skin-fat interface and the linea alba. VFT was defined as the distance between the anterior wall of the aorta and the posterior aspect of the rectus abdominis muscle perpendicular to the aorta [20]. The intra-observer technical error of measurement for the VFT was between 1.4-2.3% and 1.1-1.7% for the SFT.

The bilateral common carotid arteries were scanned for IMT measurement by using the SSA-660A (Toshiba) with a 12-MHz linear transducer. The intima-media thickness was defined as the distance between the media-adventitia interface and the lumen-intima interface. Measurements of CIMT were conducted at three differential plaque free sites: the point of the greatest thickness and 1 cm upstream and 1 cm downstream of that point. The mean the three measurements of the right and left IMT was defined as the mean IMT [22].

### Statistical analysis

Statistical analysis was performed using SPSS 17.0 (SPSS Inc, Chicago, IL, USA). Data are reported as mean ± standard deviation (SD), median (interquartile range 25%-75%) or number of participants (percentages). Non-normally distributed variables, that is, triglyceride, hs-CRP and HOMA-IR were transformed as natural logarithms before analysis. Partial correlation analysis was performed to estimate the relationships between CIMT and VFT, SFT and other CVD risk factors adjusted for BMI. Multivariate regression analysis was performed to investigate the relationships between SAT, VAT and CIMT after adjustment for covariates including CVD risk factors. P-values <0.05 were considered statistically significant.

## Results

### Clinical characteristics of the subjects

Baseline characteristics of subjects are shown in Table 1. Of 234 patients, 131 (55.7%) were men and 103 (43.8%) were women. The mean age of all subjects was 53 years and the mean duration of diabetes was 6.4 years. One hundred and thirty four (57.3% of total, 55% of men and 60% of women) were treated for hypertension. One hundred and thirty three (57% of total) were treated with statin. Twenty-one (21% of total) were treated with thiazolidinediones. The prevalence of smoking was 20.7% (32.8% of men and 5.0% of women). The prevalence of CVD was 11.5% (13.7% of men and 8.7% of women). There were no differences between men and women in mean BMI, HbA1_C_, TC, LDL-C, hs-CRP, HOMA-IR, ABI, baPWV and CIMT. The prevalence of hypertension, prescribed medication of statin, angiotensin converting enzyme inhibitor (ACEI) or angiotensin receptor blocker (ARB), and TZD were not different between men and women. However, the men were older and tended to have lower HDL-C and higher TG. Compared to women, men had significantly higher VFT and lower SFT (41.9 mm vs. 35.2 mm, 13.8 mm vs. 15.3 mm, p = 0.002, p = 0.04, respectively).

**Table 1:** Baseline characteristics of the participants

	**Total (n = 234)**	**Men (n = 131)**	**Women (n = 103)**	**P-value**
Age (Year)	53.4 ± 12.1	52 ± 11.9	55.2 ± 12.1	0.04
BMI (kg/m^2^)	25.2 ± 3.8	25.5 ± 3.7	25.9 ± 3.9	0.22
SBP (mmHg)	125.1 ± 15.5	126.7 ± 16.1	123.1 ± 14.4	0.07
DBP (mmHg)	76.3 ± 9.7	77.3 ± 9.6	75 ± 9.7	0.07
HTN (%)	134 (57.3%)	72 (55%)	62 (60%)	0.25
DM duration (years)	6.4 ± 6.2	6.1 ± 5.9	6.7 ± 6.7	0.46
HbA1_C_ (%)	8.3 ± 2.1	8.4 ± 2.2	8.1 ± 1.9	0.39
TC (mg/dl)	170.6 ± 39.3	167.7 ± 39.6	174.2 ± 38.8	0.22
LDL-C (mg/dl)	100.8 ± 35.5	99 ± 34.5	103.1 ± 36.7	0.40
HDL-C (mg/dl)	47.5 ± 11.8	45.4 ± 11.3	50.1 ± 11.9	0.003
Triglyceride (mg/dl)	119 (83, 179)	128 (86,195)	102 (82, 156)	0.001
HsCRP (mg/dl)	0.11 (0.06-0.24)	0.1 (0.06, 0.24)	0.12 (0.06, 0.23)	0.50
HOMA-IR	3.8 (2.4, 5.8)	4.0 (2.4-6.1)	3.6 (2.3-5.2)	0.32
Mean ABI	1.14 ± 0.08	1.14 ± 0.09	1.14 ± 0.06	0.89
Mean baPWV (cm/sec)	1506 ± 313	1516 ± 320	1493 ± 304	0.59
Mean CIMT (mm)	0.48 ± 0.14	0.48 ± 0.15	0.49 ± 0.14	0.89
VFT (mm)	39 ± 16.1	41.9 ± 15.5	35.2 ± 16.2	0.002
SFT (mm)	14.5 ± 5.8	13.8 ± 6	15.3 ± 5.5	0.039
VFT/SFT Ratio	3.2 ± 2.1	3.5 ± 1.7	2.8 ± 2.4	0.01
ARB or ACEI, n(%)	94 (40.2%)	58 (44.3%)	36 (35.3%)	0.11
Statin, n (%)	133 (57%)	74 (56.5%)	59 (57.8%)	0.47
Antiplatelet agent, n (%)	105 (45%)	66 (50.4%)	39 (38.2%)	0.04
Thiazolidinediones, n (%)	21 (9%)	14 (10.7%)	7 (6.8%)	0.212
Smoking, n (%)	48 (20.7%)	43 (32.8%)	5 (5%)	<0.001
Prevalence of CVD, n (%)	27 (11.5%)	18 (13.7%)	7 (8.7%)	0.304
Prevalence of DN, n (%)	61 (26.1%)	39 (30%)	22 (21.4%)	0.073

Data are means ± SD or median (interquartile range). Triglyceride, HsCRP, Insulin, and HOMA-IR, are expressed as median (interquartile range) due to skewed distribution.

*Abbreviations*: BMI: body mass index; SBP: systolic blood pressure; DBP: diastolic blood pressure; HTN: hypertension; DM: diabetes mellitus; HbA1_C_: glycated hemoglobin; TC: total cholesterol; LDL-C: low density lipoprotein cholesterol; HDL-C: high density lipoprotein cholesterol; hsCRP: high sensitivity c-reactive protein; HOMA-IR: homeostasis model assessment-insulin resistance; ABI: ankle brachial index; baPWV: brachial ankle pulse wave velocity; CIMT: carotid intima media thickness; VFT: visceral fat thickness; SFT: subcutaneous fat thickness; ARB: angiotensin receptor blocker; ACEI: angiotensin converting enzyme inhibitor; CVD: cardiovascular disease; DN: diabetic nephropathy.

### Correlations of CIMT with VFT and SFT

In simple correlation analysis, VFT was positively associated with BMI (r = 0.55), DBP (r = 0.19), TC (r = 0.21), TG (r = 0.35), and insulin (r = 0.23), and negatively associated with CIMT (r = -0.19) in men. VFT was positively associated with BMI (r = 0.77), hs-CRP (r = 0.33), HbA1C (r = 0.30), and TG (r = 0.27), and negatively associated with HDL-C (r = -0.34) in women. SFT showed positive correlations with BMI (r = 0.53) and insulin (r = 0.27) and negative correlations with CIMT (r = -0.33), baPWV (r = -0.25), age (r = -0.47), and duration of diabetes (r = -0.18) in men. SFT showed positive correlations with BMI (r = 0.54), hs-CRP (r = 0.24), and TC (r = 0.20) and negative correlations with duration of diabetes (r = -0.25) in women (data not shown). Table 2 shows partial correlation coefficients between CIMT, abdominal fat thickness and other clinical variables adjusted for BMI. After adjustment for BMI, in men, whereas VFT was not correlated with CIMT, and SFT was negatively correlated with CIMT (r = -0.15, p = 0.18, r = -0.27, p = 0.03, respectively). In women, VFT and SFT were not correlated with CIMT (r = -0.1, p = 0.4, r = -0.01, p = 0.93, respectively). VFT/SFT ratio was not correlated with CIMT in either men or women (data not shown). VFT showed a negative correlation with HDL-C and SFT showed a positive correlation with HDL-C in women. However, there were no correlations between abdominal fat thickness and insulin, HOMA-IR, mean baPWV and ABI after adjustment for BMI.

**Table 2:** Partial correlation coefficients of abdominal fat thickness with various variables in T2DM patients

	**VFT**				**SFT**			
	**Men**		**Women**		**Men**		**Women**	
	**r**	**p**	**r**	**p**	**r**	**p**	**r**	**p**
Age	0.23	0.05	0.17	0.22	-0.35	<0.01	-0.21	0.11
SBP	0.13	0.30	0.10	0.46	-0.03	0.82	-0.15	0.26
DBP	0.04	0.73	0.14	0.30	-0.05	0.67	-0.05	0.71
DM duration	0.03	0.79	-0.05	0.75	-0.02	0.86	-0.24	0.08
HbA1_C_	-0.16	0.20	-0.04	0.76	-0.01	0.94	-0.07	0.60
TC	0.03	0.80	0.02	0.85	0.14	0.27	0.18	0.18
LDL-C	0.04	<0.01	0.04	0.76	0.21	0.08	0.13	0.35
HDL-C	-0.16	0.20	-0.31	0.02	-0.05	0.70	0.38	0.01
TG	0.18	0.15	0.13	0.35	0.02	0.89	-0.06	0.67
HsCRP	0.16	0.07	-0.14	0.60	-0.01	0.88	-0.06	0.65
Insulin	0.125	0.31	-0.176	0.20	0.003	0.98	-0.17	0.23
HOMA-IR	-0.05	0.64	-0.17	0.21	-0.05	0.71	-0.14	0.30
CIMT	-0.15	0.18	-0.10	0.40	-0.27	0.03	-0.01	0.93
ABI	-0.11	0.39	-0.02	0.97	-0.21	0.08	-0.06	0.64
baPWV	0.19	0.12	0.02	0.89	-0.13	0.29	-0.15	0.29
VFT	-	-	-	-	-0.06	0.65	-0.05	0.71
SFT	-0.06	0.65	-0.06	0.65	--	-	-	-
VFT/SFT ratio	0.68	<0.001	0.41	<0.01	-0.59	<0.01	-0.55	<0.01

*Abbreviations*: BMI: body mass index; SBP: systolic blood pressure; DBP: diastolic blood pressure; DM: diabetes mellitus; HTN: hypertension; HbA1_C_: glycated hemoglobin; TC: total cholesterol; LDL-C: low density lipoprotein cholesterol; HDL-C: high density lipoprotein cholesterol; hsCRP: high sensitivity c-reactive protein; HOMA-IR: homeostasis model assessment-insulin resistance; ABI: ankle brachial index; baPWV: brachial ankle pulse wave velocity; CIMT: carotid intima media thickness; VFT: visceral fat thickness; SFT: subcutaneous fat thickness.

### Associations between SFT, VFT and CIMT as determined by multiple linear regression analysis

We performed multiple regression analysis to further investigate the independent associations between SFT and CIMT (Table 3). To better assess the effect of gender on the association between SFT and CIMT, an interaction (gender*SFT) was evaluated first. The interaction was statistically significant (p = 0.014) (data not shown) and then we analyzed men and women separately. As adjusting variables, we used BMI, traditional and non-traditional CVD risk factors such as SBP, LDL-C, HDL-C, age, smoking and HOMA-IR. In addition, therapeutic interventions that have been shown to influence CIMT, such as the presence of antihypertensive drugs, statin, and thiazolidinediones, were included. Multiple regression analysis using CIMT as a dependent variable showed that SFT was a significant independent contributing factor to CIMT in men but not women (beta = -0.004, p < 0.001). VFT was not a significant factor for CIMT in either men or women (p = 0.569, p = 0.835, respectively).

**Table 3:** Relation between abdominal fat and carotid atherosclerosis as determined by multiple linear regression analysis

	**Men**			**Women**		
	**(Adjusted-R**^**2**^ **= 0.396)**			**(Adjusted-R**^**2**^ **= 0.262)**		
**Variables**	**Beta**	**95% CI**	**P-value**	**Beta**	**95% CI**	**P-value**
Age (years)	0.008	0.006 ~ 0.011	<0.001	0.007	0.004-0.009	<0.001
BMI (kg/m^2^)	0.007	-0.05 ~ 0.048	0.089	0.009	-0.004 ~ 0.021	0.175
SBP (mmHg)	0.001	0.000 ~ 0.002	0.098	0.001	-0.002 ~ 0.002	0.823
HDL-C (mg/dl)	0.001	-0.002 ~ 0.002	0.748	0.001	-0.002 ~ 0.004	0.424
LDL-C (mg/dl)	0.001	0.000 ~ 0.001	0.028	0.001	0.000 ~ 0.021	0.213
TG (mg/dl)	0.001	0.000 ~ 0.001	0.016	-0.001	0.000 ~ 0.001	0.766
HOMA-IR	0.001	-0.007 ~ 0.008	0.864	-0.006	-0.015 ~ 0.004	0.243
Smoking	-0.001	-0.050 ~ 0.048	0.971	0.005	-0.147 ~ 0.158	0.946
Treatment with statin	0.007	-0.04 ~ 0.053	0.780	0.037	-0.021 ~ 0.094	0.206
Treatment with antihypertensive agents	-0.003	-0.051 ~ 0.046	0.917	-0.036	-0.103 ~ 0.031	0.287
Treatment with TZD	-0.06	-0.135 ~ 0.016	0.122	0.017	-0.089 ~ 0.123	0.75
VFT (mm)	-0.001	-0.006 ~ 0.003	0.569	0.001	-0.006 ~ 0.007	0.835
SFT (mm)	-0.004	-0.005 ~ -0.002	<0.001	-0.002	-0.005 ~ 0.001	0.175

*Abbreviations*: BMI: body mass index; SBP: systolic blood pressure; HDL-C: high density lipoprotein-cholesterol; LDL-C: low density lipoprotein-cholesterol; TG: triglyceride; HOMA-IR: homeostasis model assessment-insulin resistance; TZD: thiazolidinedione; VFT: visceral fat thickness; SFT: subcutaneous fat thickness.

## Discussion

In the present study, we examined the associations of VFT and SFT with carotid atherosclerosis in patients with T2DM, and demonstrated that SFT was inversely associated with CIMT after adjustments for BMI and other traditional or non-traditional CVD risk factors only in men and not in women. We found that there was a significant gender effect on the relationship between SFT and CIMT. Also, this study showed that VFT and VFT/SFT ratio were not significantly correlated with CIMT in both men and women.

Recent studies have shown that regional adiposity of the body is more closely associated with atherosclerosis than the magnitude of generalized obesity [1,3,23]. In particular, VAT confers risks of metabolic and CV complications [4,24,25]. The possibility of an active metabolic role for VAT in the etiology of MS and CVD may be explained by the fact that the VAT has higher lipolytic activity and directly releases free fatty acids into the portal circulation, and this considerably contributes to insulin resistance [26]. Khashper et al. reported that VAT thickness measured by CT scans were correlated with the presence and extent of coronary atheroma in asymptomatic subjects with DM [27]. Wang et al. showed that subjects with visceral fat areas ≥80 cm^2^ on MRI had significantly higher CIMT than those without abdominal obesity regardless of BMI in a sample of middle-aged Chinese men [28]. Kawamoto et al. found that maximal preperitoneal fat thickness assessed by US was an important risk factor for CIMT in patients aged ≥50 years with BMI ≥23 kgm^-2^[20]. One study performed in a sample of 368 men with T2DM demonstrated that subjects with VFT ≥47.6 mm by US, regardless of normal waist circumference (WC), had higher CIMT compared with those with increased WC, but had less visceral fat [19]. In another study by Dahlen et al., sagittal abdominal diameter evaluated by US was more important in predicting subclinical atherosclerosis, compared with WC, in middle aged patients with type 2 diabetes [29]. However, the studies noted above did not evaluate the relationship between SAT and CIMT. In contrast with these studies, our findings indicate that VFT is not associated with CIMT in either male or female patients with T2DM. Although VFT was correlated with CIMT in men, this correlation disappeared after adjustment for BMI. Because the effects of VFT or SFT on carotid atherosclerosis vary according to categories of BMI representing general adiposity, we adjusted for BMI in our statistical analyses.

Profound endocrine and metabolic activity differences exist between visceral adipose tissue and subcutaneous adipose tissue [4]. It is unclear whether subcutaneous fat tissue may be risk enhancing or, rather, protective for CVD. Some studies have revealed that the roles of subcutaneous abdominal fat in CVD risk factors or atherosclerosis are nearly identical to those for visceral abdominal fat, whereas other studies showed that subcutaneous abdominal fat may be “less pathogenic” or protective for CVD [6-9]. Subcutaneous adipose tissue displays less lipolytic activity, releases fewer inflammatory adipocytokines and has greater adiponectin gene expression than visceral adipose tissue [4]. Subcutaneous adipose tissue is a preferential storage site of energy and, therefore, subcutaneous adipose tissue has been regarded as protective fat. Subcutaneous adipose tissue identified as “protective” fat depots in previous studies were mainly in non-abdominal adipose tissue regions, such as gluteal, thigh, or leg region SAT [5,30,31]. Snijder et al. suggested a protective role for peripheral subcutaneous adipose tissue, observing that increased thigh fat mass is associated with lower glucose, and lipid levels, independent of abdominal fat [5]. In study by Park et al., thigh circumference was inversely associated with CIMT in both men and women and calf circumference was negatively correlated with CIMT in women with type 2 diabetes [31]. However, one study found that subcutaneous fat accumulation, as assessed by increased hip circumference, was associated with an increased risk of T2DM and to a degree that generally similar to an increase BMI and WC. This result indicates that subcutaneous fat does not always “protect” against metabolic diseases [30]. It is possible that, if positive caloric balance results in an increase in fat weight gain, the absolute direction of adipose tissue expansion may not differ between visceral adipose tissue versus subcutaneous adipose tissue.

In addition, some reports suggest that the effects of SAT on CVD risk may depend on accompanying amounts of VAT [30,32,33]. Few studies have assessed potential interactions between SAT and VAT. Some authors have reported that VAT may contribute to or exacerbate the pathogenic potential of SAT, such as an increase in insulin resistance, especially in obese individuals [30,32]. On the other hand, others suggest that when there are low amounts of both VAT and SAT, the effects of lipolytic and adipokine activity by SAT on cardiometabolic risk factors are more evident. As a result, the adverse effects of SAT lessen with increasing amounts of VAT [33]. Wildman et al. examined whether VAT modifies the effects of SAT assessed by CT on CIMT in 500 European- and African-Americans. They found that when there were low amounts of VAT, more SAT was associated with a higher CIMT, whereas when there were high amounts of VAT, more SAT was associated with a lower CIMT [33]. These discrepant findings regarding the role of SAT on subclinical atherosclerosis or CVD risk factors in the literature may be due to differences in VAT amounts between participants in the previous studies. However, the mechanism underlying the potential beneficial effects of SAT in the presence of high amounts of VAT is not yet clear. Our results showed that SFT was negatively associated with CIMT in men despite adjustments for VFT and BMI. To our knowledge, few studies have focused on the measurement of SFT by US in patients with T2DM. Furthermore, no published studies have evaluated the role of SAT by US for carotid atherosclerosis in patients with T2DM.

Recently, a few studies have reported that the ratio between these two abdominal fat types (VAT:SAT) is an important tool for expressing the fat distribution in the abdomen. The ratio is strongly related to atherosclerotic risk factor and CIMT in non-obese men after adjustment of various risk factors [26]. However, the respective relationships of VFT and SFT with CIMT were not significant. In disagreement with those results, our results showed that the VFT/SFT ratio was not correlated with CIMT. Our results are in agreement with those of Yamamoto et al. [34], who reported that in 98 non-obese men, the ratio of maximum thickness of the preperitioneal fat and the minimum thickness of the subcutaneous fat, was not correlated with CIMT [35].

The standard method of measurement of abdominal fat is CT or MRI [10]. However, growing evidence reveals that US is a noninvasive and reliable method to estimate these two fat compartments. Its validity compared to CT or MRI has been tested in diverse groups including older individuals, obese adults and patients with T2DM [11-13,25]. To the best of our knowledge, only two studies have investigated the differential associations of VFT and SFT measured by US with subclinical atherosclerosis in T2DM, until now [13,19]. In agreement with other studies, we found that VFT was correlated with several CVD risk factors such as BP, TC, and TG. However, these correlations disappeared after adjustment for BMI. In the present study, VFT was not correlated with HOMA-IR representing insulin resistance.

Regional fat distribution differs by gender. Previous studies reported that there are significant gender differences in abdominal adipose tissue distributions for a given waist circumference [36,37]. Consistent with other studies, we found that men have greater VFT than women and that women have thicker SFT than men, although the mean BMIs in both men and women were similar [38]. Therefore, gender must be considered when predicting abdominal fat distributions and associated subclinical atherosclerosis. We performed multiple regression analyses for men and women after adjusting for BMI, age, and other traditional and non-traditional CVD risk factors. We found that SFT was associated with CIMT only in men, irrespective of BMI.

A large proportion of patients in our study were taking antihypertensive drugs, statin, and antidiabetic drug such as thiazolidinedione, which alter the abdominal fat thickness as well as CIMT. Several antihypertensive drugs have been shown to significantly decrease CIMT [39]. Thiazolidinediones have also been shown to decrease CIMT [40]. Moreover, the data for statins have been the most impressive in regard to their effect on CIMT [41]. Therefore, we adjusted these factors in multiple linear regression analysis. However, we could not find any significant associations between presence of therapeutic medications and CIMT.

Previous data regarding the differential contributions of VAT and SAT to carodiometabolic risk factors and atherosclerosis have been conflicting. Previous studies varied in design, sample, and methods applied for the assessment of adipose tissue such as CT, MRI and US.

However, this study has several limitations. First, as this was a cross-sectional study, causal relationships between SFT and CIMT cannot be determined. Second, because our study sample included individuals who received carotid US examination for evaluation of diabetic complications, some characteristics of our sample may be substantially different from those of other samples. In addition, relatively young patients without other cardiovascular risk factors except diabetes are tend to not to be ordered carotid US, while elderly patients or those with CV risk factors are tend to be ordered US. These factors can be selection bias in this study. Third, since some of the study population had several CVD risk factors such as hypertension and dyslipidemia, we could not completely eliminate the possible effects of underlying disease or medications. Fourth, this study lacked a non-T2DM control. Therefore, the generalizability of our study may be limited. Futher prospective population-based studies are required to address this important question. Fifth, the sample size was relatively small. However, previous studies have included only about 300 subjects. A larger number of patients should be analyzed for the confirmation of our results. Sixth, we used US for the measurement of fat thickness because it was considered the best cost-effective method at this time. Although CT and MRI are more accurate methods for the measurement of fat thickness, exposure to ionizing radiation of the former and the high cost of the later limit their usage.

## Conclusions

We demonstrated that SFT assessed by US is associated with CIMT in male patients with T2DM, irrespective of BMI. To our knowledge, this is the first study demonstrating a negative association between SFT and CIMT in patients with T2DM. However, whether SFT assessed by US may be an important predictive factor for subclinical atherosclerosis in T2DM or not, further prospective studies with much larger sample sizes are needed.

## Abbreviations

VAT: Visceral adipose tissue; SAT: Subcutaneous abdominal adipose tissue; T2DM: Type 2 diabetes mellitus; US: Ultrasonography; CIMT: Carotid intima-media thickness; VFT: Visceral fat thickness; SFT: Subcutaneous fat thickness; MS: Metabolic syndrome; CVD: Cardiovascular disease; CHD: Coronary heart disease; CT: Computed tomography; MRI: Magnetic resonance imaging; HbA1c: Glycated hemoglobin; TC: Total cholesterol; LDL-C: Low density lipoprotein cholesterol; HDL-C: High density lipoprotein cholesterol; TG: Triglyceride; hs-CRP: High-sensitivity C-reactive protein; HOMA-IR: Homeostasis model assessment-insulin resistance; baPWV: Brachial ankle pulse wave velocity; ABI: Ankle brachial index; DN: Diabetic nephropathy; BMI: Body mass index; SD: Standard deviation; SBP: Systolic blood pressure; DBP: Diastolic blood pressure; ACEI: Angiotensin converting enzyme inhibitor; ARB: Angiotensin receptor blocker.

## Competing interests

We declare that there are no competing interests.

## Authors’ contributions

CHJ, BYK, KJK, SHJ, CHK, SKK and JOM contributed to the design, analysis and interpretation of this study. BYK and KJK contributed to the collection of clinical and laboratory data. BYK, CHJ and SHJ contributed to the writing of this manuscript. JOM revised the manuscript and contributed to the discussion. All authors read and approved the final manuscript.

## Authors’ information

Chan-Hee Jung and Bo-Yeon Kim are first authors.
